# Ablação por Cateter como Terapia de Primeira Linha no Tratamento da Fibrilação Atrial – Devemos Sempre Indicar?

**DOI:** 10.36660/abc.20220362

**Published:** 2022-07-07

**Authors:** Luiz Eduardo Montenegro Camanho, Gustavo Vignoli dos Santos

**Affiliations:** 1 Hospital Pró-Cardíaco Serviço de Arritmias e Estimulação Cardíaca Artificial Rio de Janeiro RJ Brasil Hospital Pró-Cardíaco - Serviço de Arritmias e Estimulação Cardíaca Artificial, Rio de Janeiro, RJ – Brasil

**Keywords:** Arritmias Cardíacas/complicações, Fibrilação Atrial/complicações, Tromboembolia/mortalidade, Terapia Trombolítica/efeitos adversos, Ablação por Cateter/métodos

A fibrilação atrial (FA) é a arritmia cardíaca mais comum na prática clínica, afetando aproximadamente 1 a 2% da população em geral e está associada a um risco aumentado de eventos cardioembólicos e impacto negativo na qualidade de vida. A taxa de mortalidade cardiovascular descrita é de aproximadamente 5% no ano^1^ e estima-se que o risco de complicações cardiovasculares seja maior no primeiro ano após o diagnóstico da arritmia.^2^ A taxa de recorrência da FA sem um tratamento preventivo e adequado é da ordem de 90%, o que expressa a magnitude do problema.^3^

Desta forma, parece bastante razoável postular o conceito de que a abordagem precoce da FA traga benefícios clínicos relevantes a estes pacientes. Dados recentes obtidos do estudo EAST-AFNET^4^ demonstraram claramente que esta abordagem é uma estratégia válida e eficaz. O estudo envolveu 2789 pacientes com diagnóstico de FA há pelo menos 12 meses que foram randomizados para tratamento precoce da FA (ablação: 8% e DAA: 87%) ou tratamento conservador. Em um período de acompanhamento médio de 5,1 anos, o grupo de tratamento precoce demonstrou uma redução significativa no desfecho primário de morte cardiovascular em relação ao grupo conservador. O risco de AVC, hospitalização por IC ou síndrome coronariana aguda também foi menor no grupo de abordagem precoce. O desenho do estudo não se propôs primariamente avaliar segurança e efetividade dos componentes do tratamento precoce (ablação vs. drogas antiarrítmicas - DAA). Sendo assim, os autores concluíram que a estratégia de controle precoce do ritmo cardíaco se associou a um menor risco de desfechos desfavoráveis do que o tratamento usual em portadores de FA e condições cardiovasculares associadas.

A ablação por cateter já se mostrou uma alternativa superior ao tratamento farmacológico quanto ao controle do ritmo e melhora da qualidade de vida.^5–7^ Vários ensaios anteriores também já demonstraram o claro benefício da ablação por cateter da FA como terapia de primeira linha, reforçando o conceito de que um menor tempo do diagnóstico à ablação está associado a uma menor taxa de recorrência e menor número de procedimentos repetidos, além da redução da hospitalização.^8,9^ De maneira similar, o menor tempo entre o primeiro diagnóstico de FA persistente à ablação reduz a ocorrência de gatilhos extra veias pulmonares e recorrência de taquiarritmias atriais.^10^

Nesta revista, Cardoso et al.,^11^ apresentaram uma elegante revisão sistemática e metanálise sobre a superioridade da ablação por cateter como terapia de primeira linha em relação as DAA para FA.

Os ensaios selecionados deveriam preencher todos os seguintes critérios de inclusão: ensaios controlados randomizados de ablação por cateter vs. DAA; pacientes com FA que não receberam tratamento com DAA; análise de quaisquer dos seguintes desfechos de interesse: recorrência de taquicardia atrial, recorrência de FA sintomática, hospitalizações, bradicardia sintomática, e qualidade de vida. Os critérios de exclusão foram estudos não randomizados, ensaios incluindo pacientes submetidos previamente à ablação por cateter ou à terapia com DAA sem sucesso.

Inicialmente foram identificados 1281 estudos pela estratégia de busca e, ao final, foram incluídos 5 estudos, com 994 pacientes, dos quais 502 (50,5%) foram submetidos à ablação por cateter, com um tempo de acompanhamento que variou de um a cinco anos.

A recorrência de TA foi significativamente menos frequente nos pacientes tratados com ablação por cateter (147/ 502; 29,2%) em comparação à DAA (245/492; 49,8%) (OR 0,36; IC95% 0,25-0,52; p < 0,001). A recorrência de FA sintomática também foi menor no grupo da ablação por cateter (57/398; 14,3%) em comparação ao grupo das DAA (118/393; 30%), assim como a taxa de internações hospitalares (21/436; 4,8% vs. 66/431; 15,3%) (OT 0,25; IC95% 0,15-0,42; p<0,001). A bradicardia sintomática não foi diferente entre os dois grupos (OR 0,55; IC95% 0,18-1,65; p=0,28). Derrame ou tamponamento cardíaco ocorreu em 8/ 464 pacientes no grupo da ablação (1,7%).

Os autores então concluem que os achados obtidos desta revisão sistemática sugerem maior eficácia da ablação por cateter como estratégia inicial de controle do ritmo cardíaco em pacientes com FA sintomática.

Dois recentes e importantes estudos lançaram luz sobre este tema, o EARLY-AF e o STOP-AF.^12,13^ Ambos utilizaram a técnica de crioablação e demonstraram claramente a superioridade da ablação por cateter em relação as DAA como terapia de primeira linha na abordagem destes pacientes.

Como se pode observar, o benefício desta estratégia tem extensa comprovação científica. No entanto, a questão de indicar sistematicamente ablação por cateter como terapia inicial antes das DAA encontra algumas limitações no mundo real: o acesso limitado dos pacientes a este tipo de intervenção; os custos envolvidos e as fontes pagadoras; a aceitação por parte do paciente; e, acima de tudo, a aceitação e incorporação desta conduta como uma prática clínica comprovadamente benéfica e segura para os nossos pacientes.
