# Supplementary material for: Ablação por Cateter como Terapia de Primeira Linha no Tratamento da Fibrilação Atrial – Devemos Sempre Indicar?
Source: Arq Bras Cardiol. 2022 Jul 7;119(1):95–6. [Article in Portuguese] doi: 10.36660/abc.20220362 (PMC9352133; doi:10.36660/abc.20220362)
Supplement: Translation

# Translation: Catheter Ablation as First-Line Therapy in the Treatment of Atrial Fibrillation – Should We Always Indicate it?

**Authors:** Luiz Eduardo Montenegro Camanho, Gustavo Vignoli dos Santos

**Affiliations:** 1 Rio de Janeiro RJ Brazil Hospital Pró-Cardíaco - Serviço de Arritmias e Estimulação Cardíaca Artificial, Rio de Janeiro, RJ – Brazil

**Keywords:** Arrhythmias, Cardiac/complications, Atrial, Fibrillation/complications, Thromboembolism/mortality, Thrombolytic Therapy/adverse effects, Catheter Ablation/methods

Atrial fibrillation (AF) is the most common cardiac arrhythmia in clinical practice, affecting approximately 1 to 2% of the general population and is associated with an increased risk of cardioembolic events and a negative impact on quality of life. The cardiovascular mortality rate described is approximately 5% per year,^1^ and it is estimated that the risk of cardiovascular complications is higher in the first year after the diagnosis of arrhythmia.^2^ The recurrence rate of AF without adequate preventive treatment is around 90%, which expresses the magnitude of the problem.^3^

Thus, it seems quite reasonable to postulate the concept that an early approach to AF brings relevant clinical benefits to these patients. Recent data obtained from the EAST-AFNET4^4^ study clearly demonstrated that this approach is a valid and effective strategy. The study involved 2789 patients diagnosed with AF for at least 12 months who were randomized to early treatment of AF (ablation: 8% and AAD: 87%) or conservative treatment. At a median follow-up period of 5.1 years, the early treatment group demonstrated a significant reduction in the primary endpoint of cardiovascular death compared to the conservative group. The risk of stroke, hospitalization for HF or acute coronary syndrome was also lower in the early approach group. The study design was not primarily intended to assess the safety and effectiveness of early treatment components (ablation vs. antiarrhythmic drugs - AAD). Therefore, the authors concluded that an early heart rhythm control strategy was associated with a lower risk of unfavorable outcomes than usual care in patients with AF and associated cardiovascular conditions.

Catheter ablation has proved to be a superior alternative to pharmacological treatment in rhythm control and improved quality of life.^5–7^ Several previous trials have also demonstrated the clear benefit of catheter ablation of AF as first-line therapy, reinforcing the concept that a shorter time from diagnosis to ablation is associated with a lower rate of recurrence and fewer repeat procedures and a reduction in hospitalization.^8,9^ Similarly, the shorter time from the first diagnosis of persistent AF to ablation reduces the occurrence of extrapulmonary vein triggers and recurrence of atrial tachyarrhythmias.^10^

In this journal, Carddoso et al.^11^ presented an elegant systematic review and meta-analysis on the superiority of catheter ablation as first-line therapy over AADs for AF.

Trials selected should meet all of the following inclusion criteria: randomized controlled trials of catheter ablation vs. AAD; AF patients who did not receive AAD treatment; analysis of any of the following outcomes of interest: recurrence of atrial tachycardia, recurrence of symptomatic AF, hospitalizations, symptomatic bradycardia, and quality of life. Exclusion criteria were non-randomized studies and trials, including patients who had previously undergone catheter ablation or AAD therapy without success.

Initially, 1281 studies were identified by the search strategy, and, in the end, 5 studies were included, with 994 patients, of which 502 (50.5%) underwent catheter ablation, with a follow-up time that ranged from one to five years old.

The recurrence of AT was significantly less frequent in patients treated with catheter ablation (147/502; 29.2%) compared to AAD (245/492; 49.8%) (OR 0.36; 95%CI 0.25 -0.52; p<0.001). Recurrence of symptomatic AF was also lower in the catheter ablation group (57/398; 14.3%) compared to the AAD group (118/393; 30%), as was the rate of hospital admissions (21/436; 4.8% vs. 66/431; 15.3%) (OT 0.25; 95% CI 0.15-0.42; p<0.001). Symptomatic bradycardia was not different between the two groups (OR 0.55; 95%CI 0.18-1.65; p=0.28). Effusion or cardiac tamponade occurred in 8/464 patients in the ablation group (1.7%).

The authors then conclude that the findings obtained from this systematic review suggest greater efficacy of catheter ablation as an initial strategy to control heart rhythm in patients with symptomatic AF.

Two recent and important studies shed light on this topic, the EARLY-AF and the STOP-AF.^12,13^ Both used the cryoablation technique and clearly demonstrated the superiority of catheter ablation over AADs as first-line therapy in managing these patients.

As can be seen, the benefit of this strategy has extensive scientific evidence. However, the question of systematically indicating catheter ablation as initial therapy before AAD encounters some limitations in the real world: patients’ limited access to this type of intervention; the costs involved and the sources of payment; patient acceptance; and, above all, the acceptance and incorporation of this conduct as a clinical practice proven to be beneficial and safe for our patients.
